# Toward a hybrid brain-computer interface based on repetitive visual stimuli with missing events

**DOI:** 10.1186/s12984-016-0179-9

**Published:** 2016-07-26

**Authors:** Yingying Wu, Man Li, Jing Wang

**Affiliations:** Department of Instrument Science and Technology, School of Mechanical Engineering, Xi’an Jiaotong University, Xi’an, ShaanXi China

**Keywords:** Brain Computer Interface (BCI), Electroencephalogram (EEG), Steady-state visually evoked potential (SSVEP), Omitted stimulus potential (OSP)

## Abstract

**Background:**

Steady-state visually evoked potentials (SSVEPs) can be elicited by repetitive stimuli and extracted in the frequency domain with satisfied performance. However, the temporal information of such stimulus is often ignored. In this study, we utilized repetitive visual stimuli with missing events to present a novel hybrid BCI paradigm based on SSVEP and omitted stimulus potential (OSP).

**Methods:**

Four discs flickering from black to white with missing flickers served as visual stimulators to simultaneously elicit subject’s SSVEPs and OSPs. Key parameters in the new paradigm, including flicker frequency, optimal electrodes, missing flicker duration and intervals of missing events were qualitatively discussed with offline data. Two omitted flicker patterns including missing black/white disc were proposed and compared. Averaging times were optimized with Information Transfer Rate (ITR) in online experiments, where SSVEPs and OSPs were identified using Canonical Correlation Analysis in the frequency domain and Support Vector Machine (SVM)-Bayes fusion in the time domain, respectively.

**Results and conclusions:**

The online accuracy and ITR (mean ± standard deviation) over nine healthy subjects were 79.29 ± 18.14 % and 19.45 ± 11.99 bits/min with missing black disc pattern, and 86.82 ± 12.91 % and 24.06 ± 10.95 bits/min with missing white disc pattern, respectively. The proposed BCI paradigm, for the first time, demonstrated that SSVEPs and OSPs can be simultaneously elicited in single visual stimulus pattern and recognized in real-time with satisfied performance. Besides the frequency features such as SSVEP elicited by repetitive stimuli, we found a new feature (OSP) in the time domain to design a novel hybrid BCI paradigm by adding missing events in repetitive stimuli.

## Background

Brain Computer Interface (BCI) is a communication technology that bypasses human’s normal output pathways of muscle and peripheral nervous, providing a direct connection between human brain and external devices. For people with severe motor disabilities such as spinal cord injury (SCI), BCI is a promising tool for communication and control [1–4]. Most of BCI researches rely on the conscious modulation of noninvasively scalp electroencephalography (EEG) to either external stimuli or internal motorsensory tasks, namely, exogenous or endogenous BCI, respectively. EEG signals including sensorimotor rhythms (SMR), P300, event-related desynchronization (ERD), event-related synchronization (ERS), and steady-state visual evoked potentials (SSVEPs) have been used to design different BCI paradigms. Specifically, SSVEP and P300-based BCI usually have the advantages of high information transfer rate (ITR), high tolerance to artifacts and robust performance across users with minimal training requirement [5–20]. SSVEPs are periodically visual cortical responses evoked by repetitive stimuli with a constant frequency [5]. In previous studies, several methods such as canonical correlation analysis (CCA), Fourier Transform (FT) and common feature analysis (CFA) were employed to extract SSVEP features in EEG [7–13], where CCA is the most commonly used. Zhang et al. proposed an improved CCA algorithm called L1-regularized multiway CCA (L1-MCCA) to better recognize SSVEP. P300 is a positive potential recorded approximately 300 ms after the stimuli. Generally, P300 can be elicited with oddball, single stimulus, and omitted-stimulus paradigms [14]. Most studies developed P300-based BCI based on oddball paradigm [15–24]. Classifiers such as linear discriminant analysis (LDA) [15–17], support vector machine (SVM) [18] and Bayesian fusion [19, 20] were proved to be efficient in P300 classification.

In the recent decade, hybrid BCIs that combing two different physiological signals have been developed to improve BCI performances. Specifically, pure hybrid BCI which incorporate two different BCI paradigms is becoming the hot topic in the field [21–34]. Ferrez et al. proposed a hybrid BCI paradigm based on motor imagery (ERD/ERS) and error-related potentials [27]. Allison [28] and Brunner [29] combined motor imagery and SSVEP to improve the accuracy of hybrid BCI system. Long et al. developed a hybrid BCI based on motor imagery and P300 to control a 2-D cursor [30]. In recent years, hybrid BCI based on SSVEP and P300 received the most attention. Edlinger et al. proposed a hybrid BCI system based on SSVEP followed by P300 [31]. P300-based BCI was used to control a virtual smart home environment, while SSVEP was implemented as a toggle switch to initiate and stop P300-based BCI. Panicker et al. carried out similar study, where SSVEP was also used as a switch of P300-based BCI [32]. Yin et al. developed a hybrid BCI speller based on the fusion of P300 and SSVEP [21, 22]. SSVEP and P300 were simultaneously elicited in this study. Specifically, they used oddball paradigm to elicit P300. Xu et al. [34] proposed a hybrid BCI speller paradigm combing P300 and SSVEP blocking feature. However, the mechanism of hybrid BCI based on SSVEP and P300 remains unclear.

On the other hand, another type of P300, elicited with omitted-stimulus paradigms, defined as Omitted Stimulus Potentials (OSP), was rarely concerned in BCI. Previous studies indicated that OSPs are event-related potentials time-locked to the absence of a stimulus in a regular series with acoustic and visual stimuli [35–37]. These “slow” OSPs were elicited with a stimulus of low frequency (<2Hz). The particular phenomenon also existed in ganglion cells of salamander and mouse [38]. However, SSVEP can be elicited only with repetitive stimulus of high frequency (>6Hz) [5, 39]. In this case, it is impossible to simultaneously elicit SSVEP and OSP. Bullock et al. reported a new omitted stimulus potential called “fast” OSP recorded in the retina of fish and reptiles [40, 41]. Later studies recorded “fast” OSPs on the human scalp EEGs, using conditioning trains of light flashes with frequency >5Hz [42]. Similar phenomenon was also reported in human beings with auditory stimuli [43]. Compared with “slow” OSPs, “fast” OSPs can be elicited without attention. Thus, it is possible to simultaneously elicit SSVEP and OSP. However, how to construct an efficiently hybrid BCI based on SSVEP and OSP, remains unknown.

In this study, we systematically investigated a hybrid BCI paradigm based on SSVEP and OSP for the first time. Repetitive visual stimuli with missing events were presented to simultaneously elicit SSVEP and OSP. Key parameters including flicker frequency, missing flicker duration, optimal electrodes, missing flicker patterns, averaging times and intervals of missing events were determined to obtain satisfied BCI performance. SSVEPs and OSPs were identified using Canonical Correlation Analysis in frequency domain and Support Vector Machine (SVM) -Bayes fusion in time domain, respectively. Finally, online experiments over nine healthy subjects validated the performance of the proposed BCI paradigm. This paper was organized into four sections with this section as introduction. The materials and methods, including paradigm design, experimental setup, feature extraction of SSVEPs and OSPs were described in "Methods" section. In "Results" section, we discussed the optimization of key parameters in the proposed paradigm and presented the online results over nine healthy subjects. Discussions and conclusions were given in "Discussion" section.

## Methods

### Stimulation paradigm

**Fig. 1:**
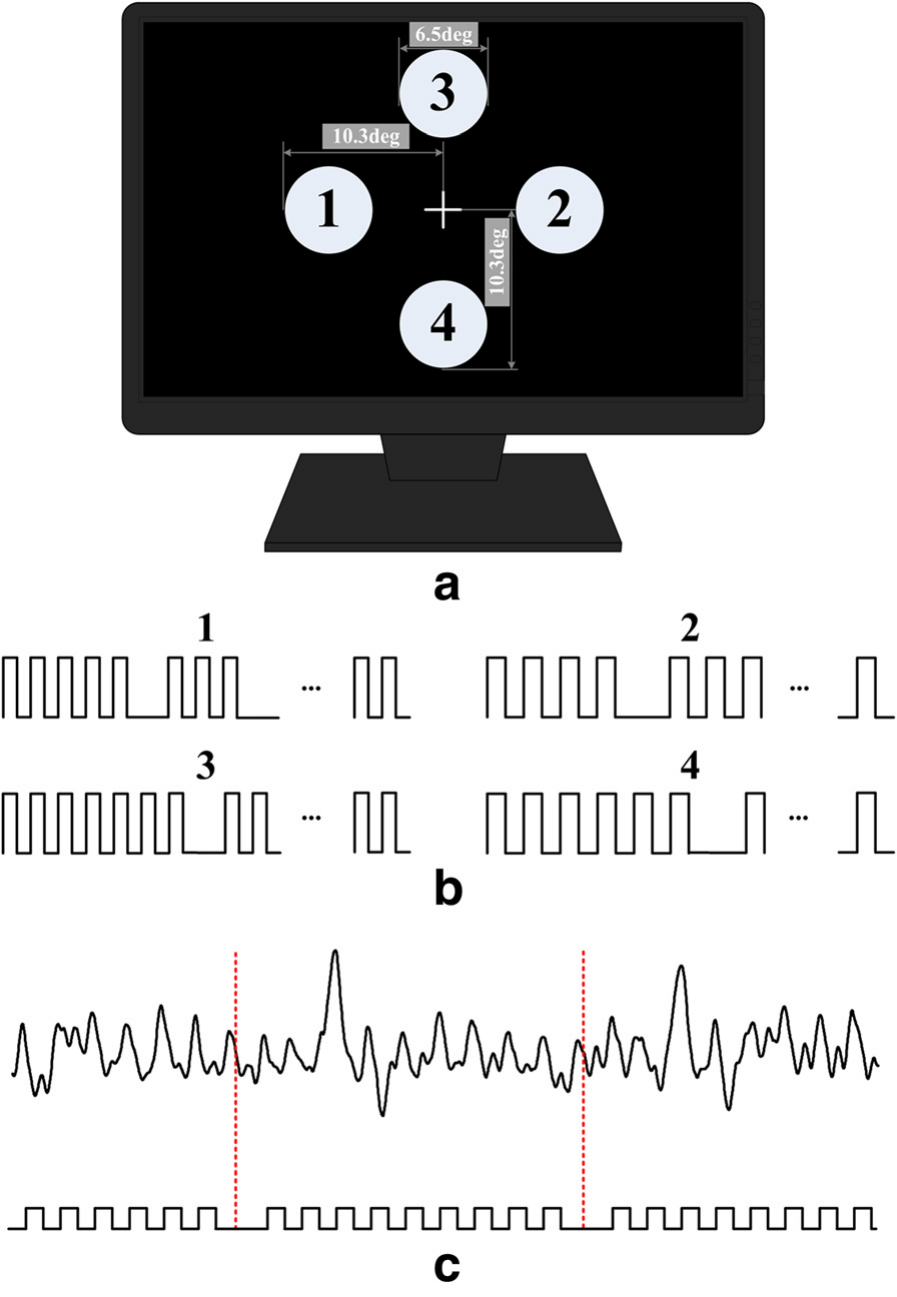
Schematic diagram of the experimental setup. **a** Distribution of four stimulators on the screen. The cross indicating the center of the monitor was not presented on the screen; **b** Stimulus sequence for four discs in **a**; **c** Typical EEG responses in the time domain to the repetitive stimuli with missing events

In this study, we proposed a novel hybrid BCI paradigm based on repetitive stimuli with missing events. It was constructed with four visual stimulators which can elicit SSVEP and OSP simultaneously and displayed on a DELL U2312HM screen with refresh rate of 60 Hz. During the experiments, the subjects’ viewing distance to the screen was 70 cm. Four stimulators were uniformly spaced in left, right, up and down directions to the center of the monitor. The stimulus luminance was 150 cd/m^2^ for the white discs and 0.7 cd/m^2^ for the black ones (Michelson contrast of 98.8 %). Each stimulator was a disc with diameter of 6.5 degrees and the distance from the outer edge of each stimulator to the center of the monitor was 10.3 degrees, as shown in Fig. 1a. Therein, disc 1 and 3 flickered at the same frequency with different onset time of missing event, so did disc 2 and 4, as shown in Fig. 1b. The high and low level of the sequence indicate white and black for the corresponding discs. Since the background color of the screen is black, “missing white discs” looks like the disc disappeared and “missing black discs” looks like the disc stopped. A typical EEG response in the time domain to the repetitive stimuli with missing events was shown in Fig. 1c. Red dotted line indicated the onset time of the missing events. Presentation of the stimulators is controlled by the Psychophysics Toolbox 3.0 [44, 45].

### Subjects and recordings

Nine right-handed healthy subjects (8 male, 1 female, age 23–26) participated in the experiments. They were requested to sit in a comfortable armchair in an ordinary lighting office room with no electromagnetic shielding. All of them had normal or corrected-to-normal eyesight and experienced BCIs before. Similar to the traditional SSVEP-based BCIs, subjects in the experiments were asked to binocularly view the screen and fixate on the center of the target stimulator.

**Fig. 2:**
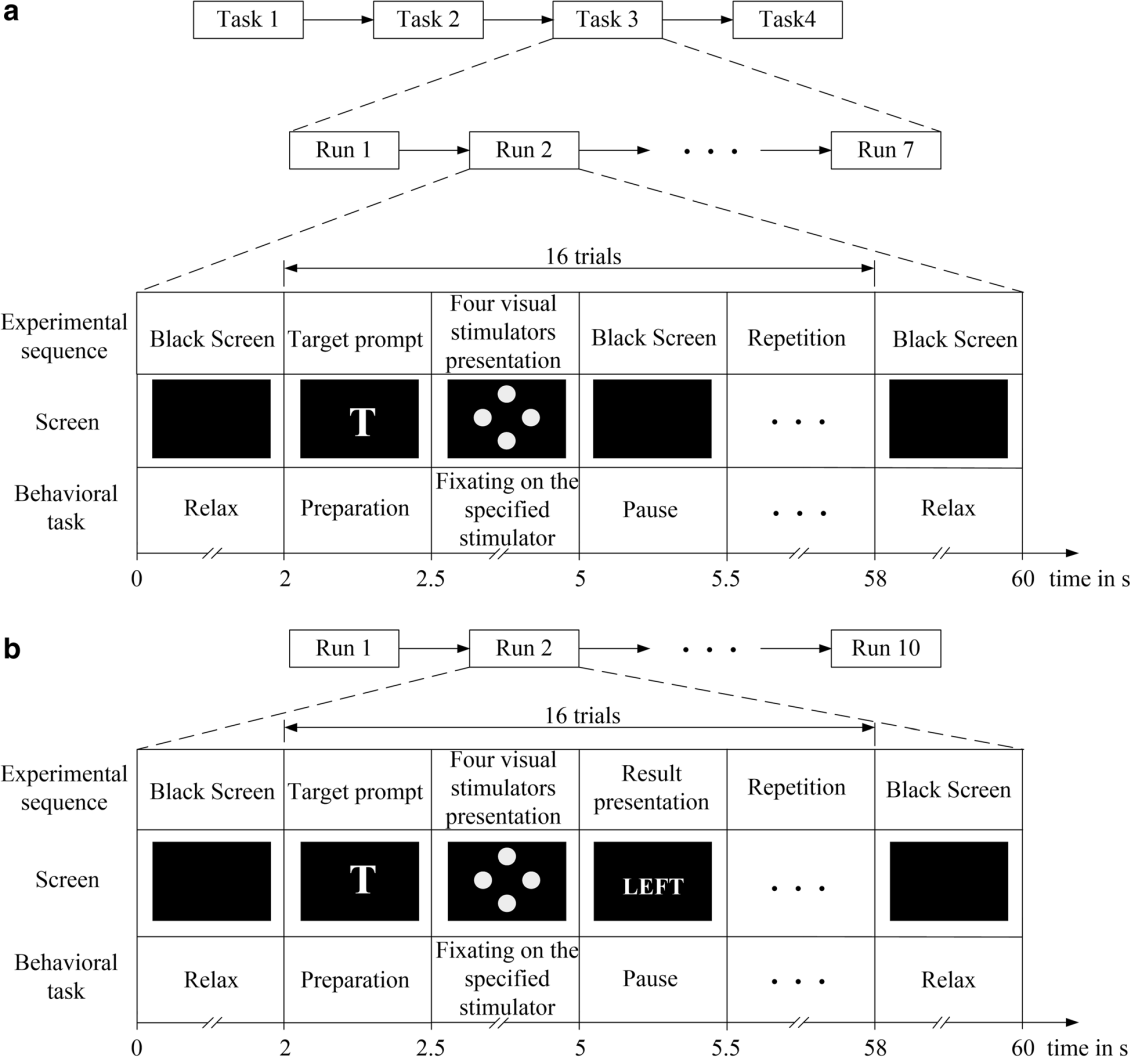
The timing of the experimental sequence and behavior task for **a** classifier training and **b** online testing

In previous SSVEP studies, EEGs were usually acquired at occipital sites [7, 46, 47]. In order to find the optimal electrodes which can best record SSVEP-OSP features in EEGs, ten electrodes including O1, O2, Oz, PO3, POz, PO4, PO7, PO8, Pz and Cz in International 10–10 System were adopted. EEGs were collected using a g. USBamp (g.tec Inc., Austria) system with sampling rate 1200 Hz. Signals were referenced to a unilateral earlobe and grounded at Fpz. Online band-pass filter of 0.01-100 Hz and notch filter between 48–52 Hz were utilized to remove artifacts and power line interference. All electrodes impedances were kept below 5 $\mathrm{k}\Omega$ during experiments. Each subject carried out the experiments composed of classifier training and online testing. During classifier training period, four experimental tasks were executed where Task 1 to Task 4 was to fixate on the four visual stimulators (disc 1–4) on the screen. Each task contained seven runs and each run included sixteen trials. Subjects were instructed to fixate on a specific stimulator throughout the task. Task 1–4 were performed one by one in random order. In each run, dark screen was first displayed for 2 s, and then the target prompt was presented for 0.5 s. After that, four stimulators were simultaneously presented for 2.5 s. Two adjacent trials were isolated by dark screen and the interval time was fixed to 0.5 s. Before the ending of each run, dark screen was displayed for 2 s and the subject was asked to relax. Each run lasted for 60 s. The classifier training period for each subject lasted for about 30 min. The timing of the experimental sequence and behavior task for classifier training was shown in Fig. 2a. In online testing, ten runs were included and each run had 16 trials. The timing of the experimental sequence and behavior task for each run in online testing (see Fig. 2b) was identical with that of classifier training period except that the classification result calculated by the trained classifier was displayed after the presentation of visual stimulators (2.5 s), which provides a feedback (success or failure) for the last task.

### Extraction of SSVEPs using canonical correlation analysis

Canonical Correlation Analysis (CCA) is a nonparametric multivariable method [48] to quantify the correlation between two signals and has been used in in extraction of SSVEPs in EEG signals. Compared with tradition methods, CCA can identify SSVEPs at satisfied accuracies without subject- specific training [49–51]. In this paper, we adopted CCA to measure the correlation between stimulus frequencies and EEGs acquired from selected electrodes. When the subject fixated on a specific stimulator, an enhanced correlation between its stimulus frequency and EEG signals was expected to occur, leading to a largest correlation coefficient among all stimulators. The acquired EEG signals were pre-processed with a band-pass filter of 1–45 Hz to remove baseline excursion and high frequency noises. Details about CCA can be referenced in our previous study [7].

### Recognition of OSPs based on Support Vector Machine (SVM) and Naive Bayes

With CCA, the flicker frequency of the target stimulator was identified. Since there are 2 stimulators flickering at the same frequency with different onset time of missing event in the proposed BCI paradigm, the target stimulator cannot be identified until the OSP features were recognized. Previous study demonstrated SVM followed by naive Bayes can solve classification problem of time series such as event related potentials (ERPs) with satisfied performance [18]. In this study, we utilize the similar scheme to detect OSP in the time domain.

#### Support vector machine

Support vector machine is an efficient tool for solving supervised classification problems due to its generalization performance and established empirical performance [52]. The basic idea of classification with SVM is to project the sample space into a high-dimensional eigenspace and find an optimal separating hyperplane (OSH) for a given feature set, which maximizes the margin between the training data and the decision boundary. The construction of OSH can be described as the following quadratic optimization problem:

1 $$ \underset{\mathbf{w}}{ \min}\frac{1}{2}\left({\mathbf{w}}^T\mathbf{w}\right)\kern0.7em s.t.\kern1em {d}^s\left(\left({\mathbf{w}}^T{\mathbf{x}}^s\right)+b\right)\ge 1 $$

where *d*^*s*^ ∈ {−1, 1} represents the sth desired output, **x**^*s*^ ∈ *R*^*p*^ is the sth input sample of the training data set {**x**^*s*^, *d*^*s*^}_*s* = 1_^*S*^ and s is the number of training vectors. In practice, the OSH probably does not exist. Hence, the slack parameters *ξ*_*s*_ ≥ 0, *s* = 1, 2, ⋯ *S* are introduced. The optimization problem now becomes:

2 $$ \begin{array}{c}\hfill \underset{\mathbf{w},\xi }{ \min}\frac{1}{2}\left({\mathbf{w}}^T\mathbf{w}\right)+C{\displaystyle \sum_{s=1}^S{\xi}_s}\hfill \\ {}\hfill s.t.\kern1em {d}^s\left(\left({\mathbf{w}}^T{\mathbf{x}}^s\right)+b\right)\ge 1-{\xi}_s;{\xi}_s\ge 0,s=1,2,\cdots S\hfill \end{array} $$

where *C* stands for the misclassification penalty term and can be considered as the regularization parameter. A larger *C* indicates higher penalty to the training errors. By introducing Lagrange multipliers α_*s*_, the OSH is computed as a decision surface:

3 $$ f\left(\mathbf{x}\right)=\operatorname{sgn}\left({\displaystyle \sum_{s=1}^S{d}^s{\alpha}_sK\left(\mathbf{x},{\mathbf{x}}^s\right)+b}\right) $$

where sgn(⋅) ∈ {±1}, x are support vectors and *K*(⋅) is the kernel function. Here, we employ the linear function defined as:

4 $$ K\left(\mathbf{x},{\mathbf{x}}^s\right)=x\cdot {x}^s $$

The regularization parameter *C* was optimized using 10-fold cross-validation. SVM was then trained using the regularization parameter *C* with the best validation performance.

### Naive bayes

Naive Bayes is a probabilistic classifier based on Bayes theorem with strong independence assumptions between the features. It is widely used in EEG classification and has satisfied performance without large amount of training samples [53]. In Bayes theorem, posteriori probability is calculated based on the priori probability:

5 $$ P\left(C\Big|A\right)=P\left(A\Big|C\right)P(C)/P(A) $$

where *A* is the set of attributes {*A*_1_, *A*_2_, ⋯, *A*_*n*_} and *C* is the set of hypotheses {*c*_1_, *c*_2_, ⋯, *c*_*m*_}. The classifier output is given by

6 $$ {c}_{nb}= \arg \underset{c_i\in C}{ \max }P\left({c}_i\Big|A\right) $$

According to Bayes theorem, it can be written as

7 $$ {c}_{nb}= \arg \underset{c_i\in C}{ \max}\left[P\left(A\Big|{c}_i\right)P\left({c}_i\right)/P(A)\right] $$

Due to the independence assumptions of the attributes, the joint probability can be calculated with

8 $$ P\left(A\Big|{c}_i\right)={\displaystyle \prod_{j=1}^nP\left(A{}_j\Big|{c}_i\right)} $$

Thus the output of Naive Bayes is:

9 $$ {c}_{nb}= \arg \underset{c_i\in C}{ \max}\frac{{\displaystyle \prod_{j=1}^nP\left(A{}_j\Big|{c}_i\right)}P\left({c}_i\right)}{P(A)} $$

### Recognition of OSPs

**Fig. 3:**
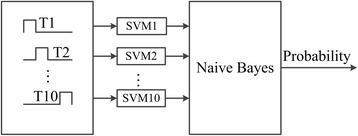
Flowchart of OSP recognition using SVM and Naive Bayes

Previous study demonstrated that OSP occurred 125–375 ms after the onset of missing events [42]. Since OSP is time-locked relative to the missing events, it will be strengthened in case that the signal is superimposed and averaged with starting line at the onset time of target stimulus. For non-target stimulus, OSPs will be weakened or even lost after averaging. The averaged segments are input into SVM and Naive Bayes to recognize targeted OSPs with FieldTrip toolbox [54], as shown in Fig. 3.

In time windows *T* = {*Τ*_1_, …, *Τ*_10_} = {{125*ms* − 150*ms*}, …, {350*ms* − 375*ms*}}, EEG signals acquired from electrodes *L* = {*O*1, *Oz*, *O*2, *POz*, *PO*4, *PO*8} construct a feature sets

*x*_*i*_(*L*, *T*_*i*_) = [*x*(*l*_1_, *T*_*i*_), …, *x*_*i*_(*L*, *T*_*i*_) = [*x*(*l*_1_, *T*_*i*_), …, *x*(*l*_6_, *T*_*i*_)] *i* = 1, 2, …, 10 where *x*_*i*_*(l,T*_*i*_*)* stands for the EEG segment in time window *T*_*i*_ at electrode *l*. The feature sets are input into SVMs to obtain the classification results *d*_*k*_*,k* = 1,2,…,10. The results are further input into Naive Bayes to get the probability of fixation on a specific stimulator:

10 $$ P={\displaystyle \prod_{k=1}^{10}{p_{k01}}^{\left(1-{d}_k\right)}{p_{k11}}^{d_k}}/{\displaystyle \prod_{k=1}^{10}{p_{k00}}^{\left(1-{d}_k\right)}{p_{k10}}^{d_k}} $$

Where $$ \begin{array}{cc}\hfill {p}_{kmn}=p\left({d}_k=m\left|c=n\right.\right)\hfill & \hfill m,n=0,1\hfill \end{array} $$. *c* = 1 indicates the target stimulator. *p*_*k*01_, *p*_*k*11_, *p*_*k*00_, *p*_*k*10_ is obtained during classifier training. When the classifier is performed on the segments superimposed with different onset time of missing events, the target stimulator with maximum probability of fixation *P* is recognized. As shown in Fig. 2a, each subject was asked to perform 4 tasks (fixating on the four visual stimulators) during classifier training. Each task contained 7 runs and each run included 16 trials. So, there were 6 × 4 × 7 × 16 = 2688 samples during classifier training for each subject. The dimensionality of each feature vector was 10 × 30.

### Information transfer rate

In order to evaluate the proposed BCI paradigm, Information Transfer Rate (ITR) was used to measure the achievable information rate per unit time, given the detection accuracy and the time required for target identification:

11 $$ \mathrm{I}\mathrm{T}\mathrm{R}=\frac{60}{DTI}\left[{ \log}_2N+Acc{ \log}_2Acc+\left(1-Acc\right){ \log}_2\left(\frac{1-Acc}{N-1}\right)\right] $$

where *N* is the number of stimulators, *Acc* is the mean detection accuracy averaged over all stimulators and *DTI* is the decision transfer interval (i.e., the sum of single detection time and interval between detections). Furthermore, one-way ANOVA was performed in SPSS19 (SPSS Inc., Chicago, Illinois, USA) for statistical comparison of system performance across all the subjects. The significance level for all statistical analyses was set at *p* < 0.05.

## Results

### Stimulus frequencies

**Fig. 4:**
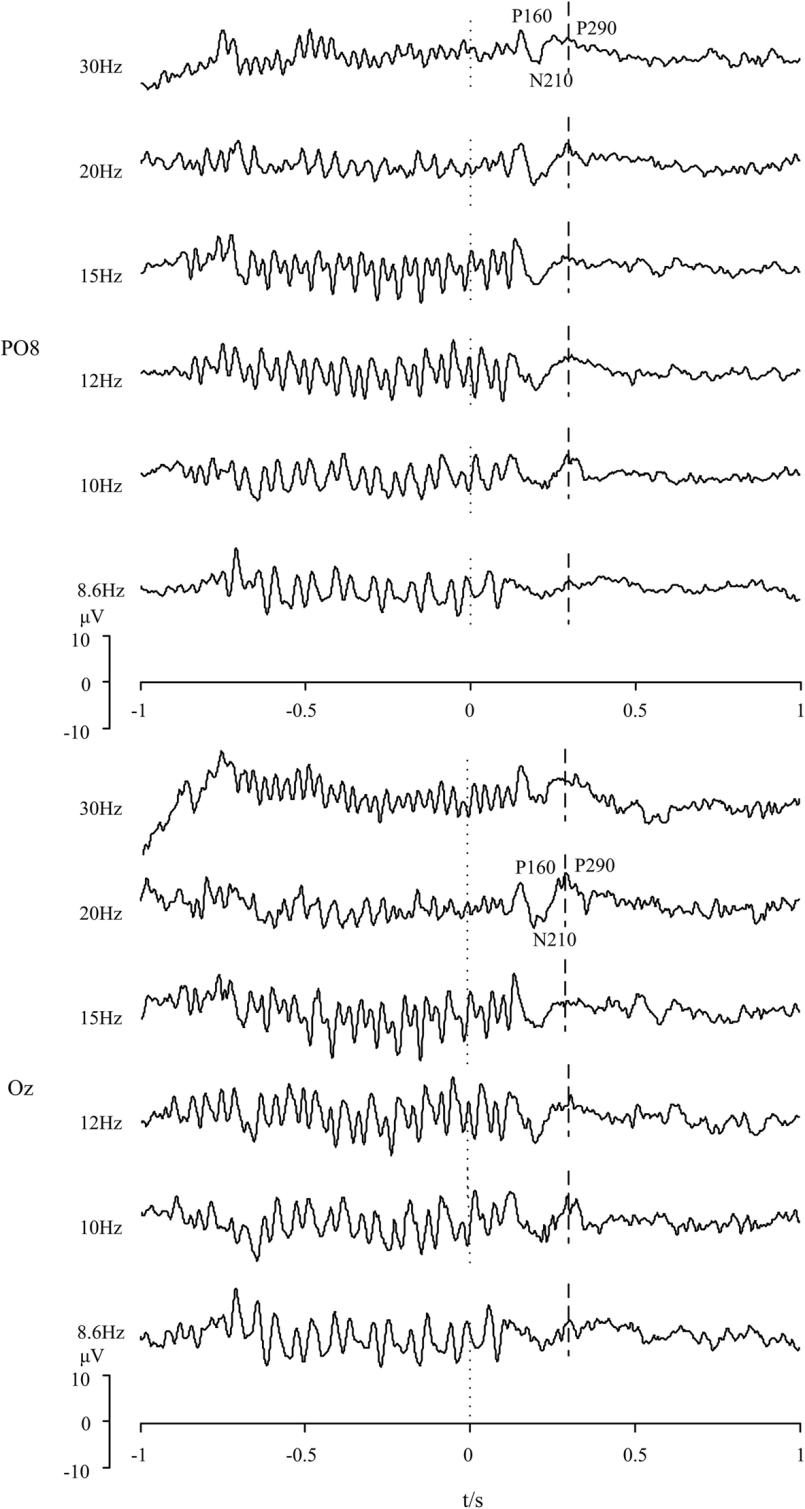
Comparison of SSVEPs and OSPs at PO8 and Oz elicited by different stimulus frequencies

Due to the limitation of refresh rate of the computer screen (60 Hz), stimulus frequencies including 30 Hz, 20 Hz, 15 Hz, 12 Hz, 10 Hz and 8.6 Hz were selected to elicit SSVEP-OSP features. In order to clarify the frequency effect, one disc flickered at different frequencies for 1 s followed by absence of black disc (namely, the disc was paused on white) for another 1 s. Subjects were asked to decrease eye-blinking frequency and avoid body movements during stimulus presentation. EEGs including 1 s before and after onset of missing flickers were superimposed and averaged over 16 trials. Typical results of two electrodes at occipital sites (PO8 and Oz) are shown in Fig. 4. Moments “0” of x-axis indicate the onset time of missing flickers (absence of black disc). For both electrodes across all stimulus frequencies, OSP features, i.e., three transient components of P1, N2 and P2 with latencies of 160, 210 and 290 ms after onset of missing flickers can be observed. Specifically, positive peak of OSP at P2 has higher amplitude with stimulus frequencies of 10 to 20 Hz, which is consistent with previous study [42]. On the other hand, stronger SSVEP responses appear at stimulus frequencies of 8.6, 10, 12 and 15 Hz. Previous studies demonstrated that SSVEP responses can be also observed at the second harmonic frequencies [7]. It would lead to confusion of SSVEP in case we select 10 and 20 Hz as the stimulus frequencies in the proposed paradigm. As a result, 10 and 12 Hz were chosen because both SSVEP and OSP features are prominent enough to be extracted in the time or frequency domain.

### Optimal electrodes

**Fig. 5:**
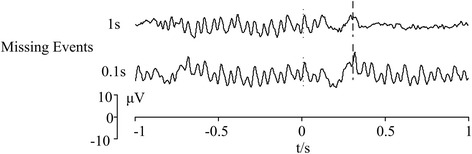
Comparison of SSVEP-OSP at O2 with different duration of missing events

Previous studies demonstrated that SSVEPs are prominent at occipital sites [7, 46, 47]. In order to locate the optimal electrodes for OSP feature, the visual stimulator was presented at frequency of 12 Hz with white disc missing for 1/12 s. Time series and topographies of EEGs after the onset of omitted stimulus are shown in Fig. 5. Specifically, OSPs with higher amplitude was recorded at O1, Oz, O2, POz, PO4 and PO8 and thus selected as optimal electrodes to extract OSPs.

### Duration of missing events

**Fig. 6:**
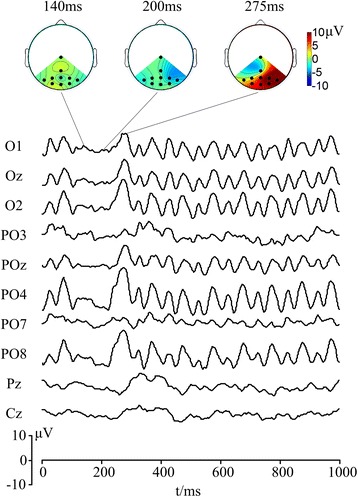
Time series and topographies of EEGs from 10-channel after the onset of omitted stimulus. Red color indicates OSPs with higher amplitude

Previous studies indicated missing events in repetitive stimuli may generate OSPs [38, 42]. Missing events with longer duration may elicit complete OSPs in the time domain whereas SSVEP features disappeared during the missing events. An ideal situation is that OSPs are elicited by missing events with very short duration (i.e., 1/Stimulus Frequency) and the negative effect to SSVEP can be minimized. In order to testify the hypothesis, repetitive stimuli with frequency of 10 Hz were used to elicit SSVEP-OSP features. The stimuli was first presented for 1 s, then paused on white for 0.1 or 1 s and presented for another 1 s. The pre-processed data (band-pass and notch filter) were superimposed and averaged over 8 single trials to extract SSVEP-OSP features. Results are shown in Fig. 6. Moment “0” of x-axis indicated the onset time of missing events. Similar SSVEP and OSP features can be elicited in both conditions. Especially, the latencies of OSP relative to the onset time of missing event (“0” of x-axis) remain the same. In the online experiment, we set the duration of missing events to minimum value (1/Stimulus Frequency), which is enough to elicit OSPs while keeping SSVEPs.

### Missing flicker patterns

**Fig. 7:**
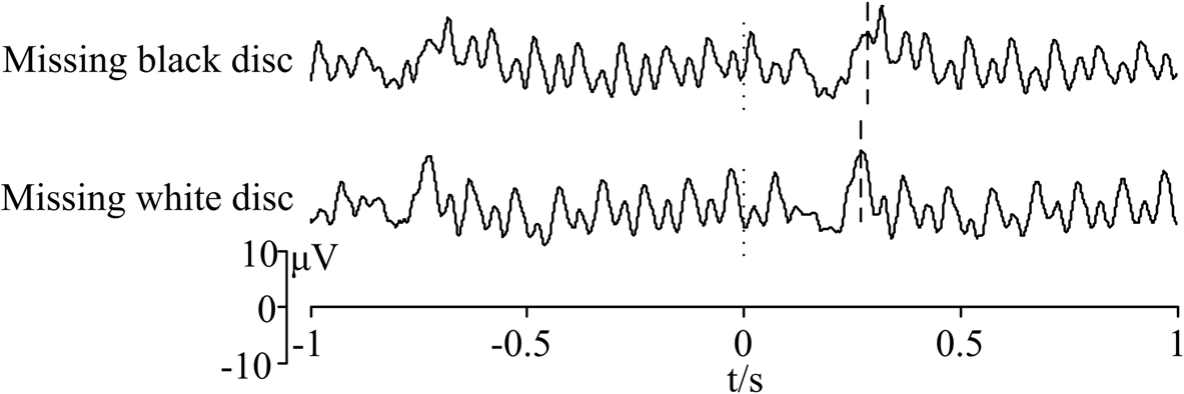
Comparison of different missing flicker patterns

Two missing flicker patterns are available in the proposed BCI paradigm: missing white and black discs. Since the background color of the screen is black during visual stimulation, “missing white discs” looks like the stimulator disappeared and “missing black discs” looks like the stimulator stopped. We qualitatively compare the SSVEP-OSP features elicited by these two missing flicker patterns. Visual stimulus was presented at frequency of 10 Hz with two different missing flicker patterns with duration of 0.1 s. EEGs 1 s before and after onset of missing pauses were superimposed and averaged over 8 trials (see Fig. 7). Moment “0” of x-axis indicated the onset time of missing flicker. Compared with missing black disc pattern, the elicited OSP with missing white disc pattern was superposed on the SSVEP with higher amplitude. Generally, OSPs can be elicited with both missing flicker patterns. Further quantitative comparisons will be made based on accuracy and information transfer rate (ITR) in online experiments.

### Interval of missing events

**Fig. 8:**
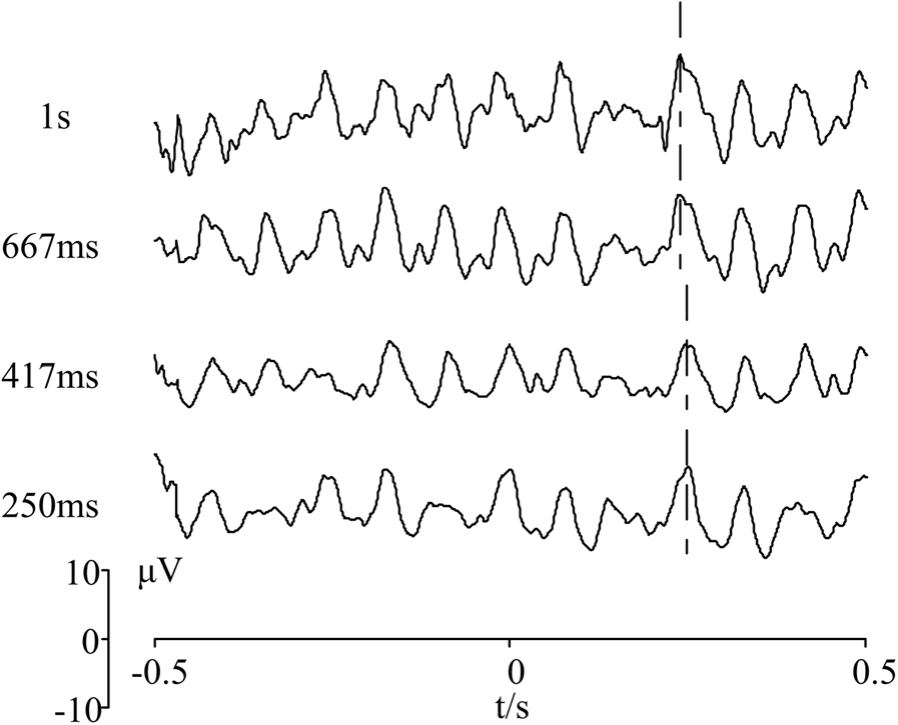
EEG responses with different interval of missing events

According to equation 11, interval of missing events affects ITR in two ways: increasing interval of missing events may (1) result in longer DTI with a negative effect on ITR; (2) strengthen SSVEP features and increase *Acc* with a positive effect on ITR. With stimulus frequency 12 Hz, typical EEG responses to different interval of missing events are shown in Fig. 8. Therein, interval of missing events 1 s, 667 ms, 417 and 250 ms correspond to 12, 8, 5 and 3 flickers (white-black-white), respectively. Though the amplitude of OSP as well as SSVEP features attenuated with the decrease of interval of missing events, previous studies demonstrated that OSPs can be elicited by two repetitive flickers with one missing events [38, 41, 42]. On the other hand, SSVEP features were still recognizable in the time domain. Thus, we set the interval of missing events to 3 flickers, i.e., two repetitive flickers followed by one missing events.

### BCI performances

Experiments were carried out with optimized parameters discussed above. The onset time of first missing event of disc 1, 2, 3, and 4 occurred at 467, 450, 633 and 650 ms, respectively. Previous studies reported ERPs such as P300 can be well extracted with averaging 8 times [14, 23, 32, 55]. In our study, we set the averaging times to 2, 4, 6, and 8 for both offline training and online testing. Results in Table 1 demonstrated each subject’s highest ITR under optimal averaging times and mean accuracy, with two different missing events patterns. The online accuracy and ITR (mean ± standard deviation) over nine healthy subjects were 79.29 ± 18.14 % and 19.45 ± 11.99 with missing black disc pattern, and 86.82 ± 12.91 % and 24.06 ± 10.95 with missing white disc pattern, respectively. With missing black and white disc pattern, six and seven out of nine subjects exceeded the level of 80 % mean accuracy and 15 bits/min ITR. No significant difference was found between two missing flicker patterns [Accuracy: F(1,16) = 0.727, *p* = 0.406; ITR: F(1,16) = 1.058, *p* = 0.319, one-way ANOVA].

**Table 1:** Online accuracy and ITR statistics with optimal parameters

Subjects	Averaging times	Missing black disc		Missing white disc	
		Accuracy(mean)(%)	ITR(bits/min)	Accuracy(mean)(%)	ITR(bits/min)
S1	4	99.01	38.08	99.69	39.30
S2	4	96.25	34.20	98.75	37.66
S3	6	90.18	23.68	97.50	30.72
S4	6	89.38	23.03	88.12	22.04
S5	6	86.25	20.65	96.88	30
S6	6	80	16.46	81.25	17.26
S7	6	65.63	9.03	61.16	7.21
S8	6	62.5	7.73	81.87	17.65
S9	6	44.37	2.19	77.08	14.74
Average		79.29 ± 17.11	19.45 ± 11.30	86.92 ± 12.17	24.06 ± 10.32

**Fig. 9:**
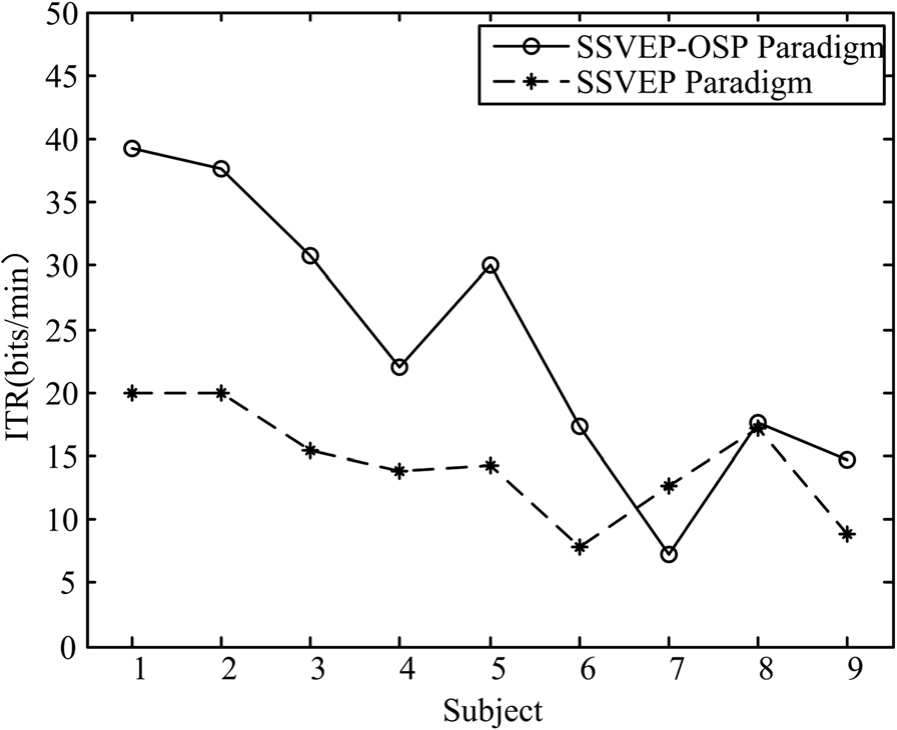
ITR comparison between SSVEP-OSP paradigm and SSVEP paradigm

In order to demonstrate the advantage of OSP to BCI performances, we further compared the proposed SSVEP-OSP paradigm and traditional SSVEP paradigm with two stimulators. The comparison is fair since both paradigms utilized two frequencies to design visual stimulators. One of the main advantages of the proposed SSVEP-OSP paradigm is that more stimulators can be presented with certain stimulus frequencies. Results shown in Fig. 9 suggested significantly higher ITR with SSVEP-OSP paradigm than that with traditional SSVEP paradigm [F(1,34) = 8.882, *p* = 0.005, one-way ANOVA]. All the subjects achieved better ITRs with SSVEP-OSP paradigm except one subject (S7).

**Fig. 10:**
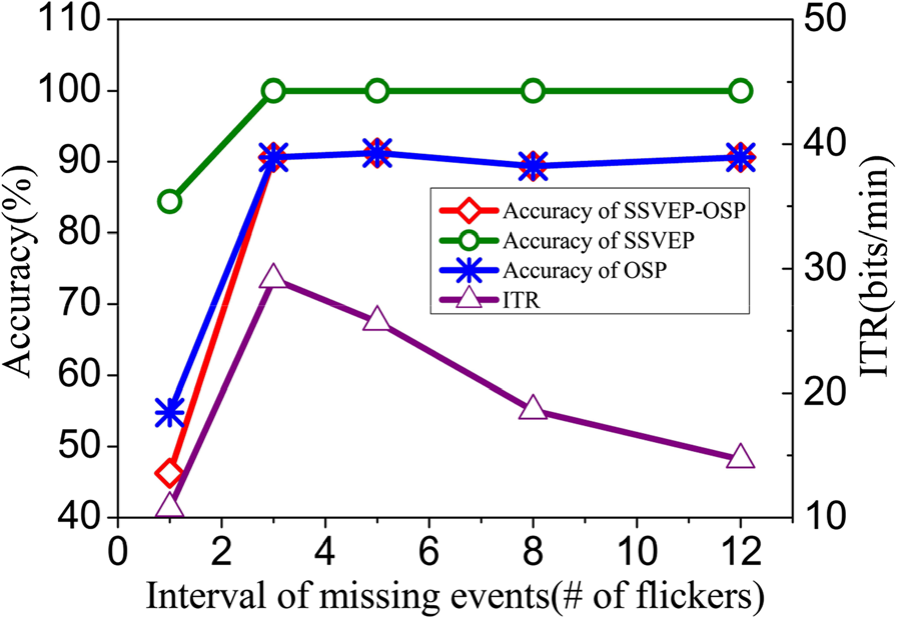
Performance with different interval of missing events

Moreover, we carried out experiments to demonstrate the effect of interval of missing events on the performance of the proposed paradigm. Interval of missing events was set to 12, 8, 5, 3 and 1 flicker(s) (white-black-white), respectively. Results were shown in Fig. 10. With increasing interval of missing events, the detection accuracy of SSVEP increased significantly (from 84.38 – 100 %). When the interval of missing events decreased to 1 flicker, the OSP pattern cannot be elicited. As a result, the detection accuracy of OSP dropped to random level (54.81 %, two stimulators) and ITR also decreased significantly (10.76 bits/min). Highest ITR (29.14 bits/min) was achieved when the interval of missing events was 3 flickers.

## Discussion

The novel hybrid BCI paradigm proposed in this study is based on simultaneous elicitation of SSVEP and OSP using repetitive visual stimuli with missing events. As suggested in previous studies, OSP is one type of P300 that elicited with omitted-stimulus paradigm [14, 56]. Here, we use “OSP” rather than “P300” for differentiation because most studies developed P300-based BCI based on oddball paradigm [5, 8, 21–24, 34, 55]. Typical hybrid BCIs based on SSVEP and oddball-P300 were proposed in [21, 22, 34, 55]. Xu et al. [34] developed a hybrid BCI paradigm combining P300 and SSVEP blocking feature. Therein, the SSVEP blocking feature is similar with the OSP in our study. However, the authors focused on the comparison of hybrid BCI and P300-BCI. The influence of several key parameters on the BCI performances, such as stimuli frequency of SSVEP and duration of SSVEP-block, was not concerned. These questions were discussed in detail in our study. Combaz et al. [55] proposed a hybrid BCI paradigm combining oddball-P300 and SSVEP. Background flickers at 12 or 15Hz were used to elicit SSVEP continuously and traditional oddball stimulus was presented 500 ms after SSVEP started. Two types of visual stimulus, i.e., repetitive stimulus with fixed frequency followed by oddball stimulus, were used to elicit SSVEP and P300 in time order, which is different from the proposed paradigm in our study. On the other hand, previous studies indicated that oddball-P300 required attention [57–59]. Fast OSPs in our study required fixation but not attention [42], which is superior to oddball-P300 paradigm. In the code-modulated visual evoked potential (c-VEP) paradigm, a pseudorandom sequence modulated by different time lag was used to construct target stimuli [60, 61]. A VEP template for the specified target stimulator can be obtained by averaging the EEG data from multiple stimulus cycles. All the target stimuli have similar VEP templates with different time lag. CCA, a template matching method in the time domain, was used for target identification. Comparatively, EEG features in both time and frequency domain were used to detect different target stimulators in our hybrid BCI paradigm, where SSVEP and OSP were identified in the frequency and time domain, respectively. One interesting point is that the VEP template in c-VEP paradigm is much more complicated than the SSVEP template in our hybrid paradigm, though CCA were used for template matching in both paradigms.

In the proposed hybrid BCI paradigm, online experiments across nine healthy subjects suggested that mean accuracy and ITR with missing black disc pattern is comparatively higher than those with missing white disc pattern, but the effect is not statistically significant [Accuracy: F(1,16) = 0.727, *p* = 0.406; ITR: F(1,16) = 1.058, *p* = 0.319, one-way ANOVA]. One possible explanation is that the elicited OSP with missing white disc pattern has higher amplitude and can be better recognized with the proposed algorithm. As a preliminary study of hybrid BCI paradigm based on SSVEP and OSP, one possible limitation is that we only present four visual stimulators on the screen, which limits the ITR of the proposed BCI paradigm. When comparing the proposed SSVEP-OSP paradigm with traditional SSVEP paradigm, we used the same time to calculate ITR for both paradigms. For subject S1 and S2, the optimal averaging times is 4, which corresponds to 2 s to output a command for both SSVEP-OSP and traditional SSVEP paradigm (1 s for visual stimulus and 1 s between two trials). For subject S3 to S9, the optimal averaging times is 6, which corresponds to 2.5 s to output a command (1.5 s for visual stimulus and 1 s between two trials). In our previous study, oscillating Newton’s rings were presented 4 s as a single trial to elicit SSVEP [7]. Other researchers also reported 1.75-4 s to detect SSVEP with satisfied performance [9–11]. So the time 2–2.5 s to output a command in the present study is fair for SSVEP comparison.

## Conclusions

In summary, we proposed a novel hybrid BCI based on SSVEP and OSP in this study. The proposed BCI paradigm is composed of four discs flickering from black to white with different stimulation frequency and/or different onset time of missing event. The online accuracy and ITR (mean ± standard deviation) over nine healthy subjects were 79.29 ± 18.14 % and 19.45 ± 11.99 bits/min with missing black disc pattern, and 86.82 ± 12.91 % and 24.06 ± 10.95 bits/min with missing white disc pattern, respectively. The main contribution of this study is to simultaneously elicit SSVEP and OSP by repetitive stimuli with missing events, and detect them in frequency and time domain in real-time, respectively. We discussed the optimal parameters in the new paradigm and proposed feasible algorithms for feature extraction and recognition. Besides the frequency features such as SSVEP elicited by repetitive stimuli in previous studies, we explored a new feature (OSP) in the time domain by adding missing events in repetitive stimuli. By adjusting the onset time of first missing events and interval of missing events, the recognizable target stimulators of the proposed hybrid BCI paradigm can be enhanced significantly. If the mean detection accuracy can maintain above 80 %, there will be large improvement on the ITR of the SSVEP-OSP paradigm. These are what we will focus on next.

## Abbreviations

BCI, brain computer interface; CCA, canonical correlation analysis; ERD, event-related desynchronization; ERS, event-related synchronization; ITR, information transfer rate; OSP, omitted stimulus potential; SMR, sensorimotor rhythm; SSVEP, steady state visually evoked potential; SVM, support vector machine
